# Prevalence, Risk Factors, and Intervention of Long-Term Sleep Disturbance After Intensive Care Unit Discharge: A Scoping Review

**DOI:** 10.7759/cureus.83011

**Published:** 2025-04-25

**Authors:** Yukari Fukamachi, Hideaki Sakuramoto, Tomoo Sato, Ryota Imanaka, Ayako Fukushima, Yohei kishi, Kan Sugishima, Gen Aikawa, Akira Ouchi

**Affiliations:** 1 Cooperative Doctoral Program in Nursing, Japanese Red Cross Kyushu International College of Nursing, Munakata, JPN; 2 Department of Nursing, Kurume University Hospital, Kurume, JPN; 3 Department of Critical care and Disaster Nursing, Japanese Red Cross Kyushu International College of Nursing, Munakata, JPN; 4 Department of Adult and Child Health Care and Acute Care Nursing, Kobe City College of Nursing, Kobe, JPN; 5 Department of Nursing, Kyorin University Hospital, Mitaka, JPN; 6 Department of Critical Care and Disaster Nursing, Japanese Red Cross Kyushu International College of Nursing, Munakata, JPN; 7 Department of Cancer Nursing, Mie University, Tsu, JPN; 8 College of Nursing, Kanto Gakuin University, Yokohama, JPN; 9 Department of Adult Health Nursing, Ibaraki Christian University, Hitachi, JPN

**Keywords:** hrqol, icu discharge, post-intensive care syndrome (pics), risk factor, sleep disturbances

## Abstract

Sleep disturbance in the intensive care unit (ICU) is common and often persistent after discharge. Sleep disturbance after ICU discharge, often classified under post-intensive care syndrome (PICS), affects health-related quality of life (HRQOL).

The aim of the current review was to map long-term sleep disturbance after ICU discharge, explore related factors, and suggest prevention and treatment measures, including those related to PICS. We searched the PubMed, CINAHL, and CENTRAL databases until June 12, 2024, using keywords related to ICU, sleep disturbance, and recovery. Inclusion criteria were the inclusion of adult patients, analysis of sleep disturbance after ICU, and evaluation of risk factors or interventions. Reviews, case reports, and non-original studies were excluded.

A total of 1,786 studies on sleep disturbance after ICU discharge were initially identified, with 52 studies ultimately included after screening. Prevalence of sleep disturbance after ICU discharge was calculated from 42 studies, with rates of 55.0% at less than one month, 49.6% at 1-3 months, 39.2% at 4-6 months, 23.2% at 7-12 months, and 15.0% over one year. Risk factors for sleep disturbance included older age, female sex, pre-hospital sleep disturbance, ICU and hospital lengths of stay, duration of mechanical ventilation, duration of sedation or analgesia, pain, delirium, and poor sleep quality during hospital ward. Long-term sleep disturbance was associated with PICS. Sleep disturbance was associated with physical disability, cognitive impairment, mental dysfunction (especially depression, post-traumatic stress disorder, and anxiety), and HRQOL, assessed at 4-6 months after hospitalization. Two randomized controlled trials (RCTs) examined interventions for sleep disturbance. Psychological counseling and a 12-month nurse-led collaborative care intervention were found effective in improving long-term sleep disturbance. On the other hand, the use of sleep aids such as earplugs and eye masks in the ICU had no such effect.

Sleep disturbance after ICU discharge is common, with over half of the patients being affected within one month, and a link to PICS symptoms exists, highlighting the need for further research on comprehensive interventions.

## Introduction and background

In the intensive care unit (ICU), while advanced medical care is provided, environmental factors such as noise and lighting [1], severity of illness [2], surgery [3], pain [4], delirium [5], anxiety [6], ventilation [7], and medication [8] lead many patients to report sleep disturbance. These environmental and medical factors significantly impact patients' sleep patterns, with reports indicating persistence even after discharge [9]. Effective interventions for sleep promotion in ICU patients include massage, aromatherapy, light/noise blocking, ventilator mode or type, earplugs or eye masks or both, relaxation interventions, foot baths, music interventions, nursing interventions, valerian acupressure, and sound masking [10]. However, it is not known whether these interventions are effective for sleep disturbances after ICU discharge.

Sleep disturbance following hospital discharge is recognized as a component of post-intensive care syndrome (PICS) [11] and has been associated with an increased risk of developing new health conditions, as well as with a decline in health-related quality of life (HRQOL) [12]. PICS is defined as “new onset or worsening of impairment(s) in physical, cognitive, and/or mental health that arose after the ICU and persisted beyond hospital discharge” [13]. However, few studies have examined the relationship between sleep disturbances and PICS following ICU discharge, and the association between long-term sleep disturbances and PICS remains unclear.

HRQOL encompasses patients’ self-reported physical, mental, and social functioning, including activities such as self-care, occupational and domestic roles, and social interactions, as well as subjective well-being, such as emotional states and symptoms like pain or fatigue, which can also be corroborated with objective assessments [14]. Long-term sleep disturbances and reductions in HRQOL, both physical and psychological, are likely to co-occur. Sleep disturbance after ICU discharge may serve as an early indicator of psychiatric disorders and therefore warrants careful evaluation [15]. To effectively reduce post-discharge sleep disturbance, it is essential to investigate its long-term associations with patient outcomes and to identify contributing ICU practices that may be modifiable.

A recent systematic review [16] revealed the incidences of sleep disturbance after discharge from critical illness as 50-66.7% (≤1 month), 34-64.3% (1-3 months), 22-57% (3-6 months), and 10-61% (≥6 months). In addition, risk factors were identified and categorized as pre-hospitalization, during hospitalization, and post-discharge factors. However, in the previous review, surgical patients and those with significant surgical issues were excluded, leaving gaps in systematically summarizing physical and therapeutic factors among critically ill patients. Additionally, no studies have addressed preventive interventions for long-term sleep disturbance initiated during ICU stay or therapeutic interventions after discharge. While some studies noted that sleep disturbance may persist up to 12 months after ICU discharge, potentially decreasing the HRQOL [15], the relationship between long-term sleep disturbance, PICS, and HRQOL remained unclear.

The aim of this scoping review was to identify and map information on long-term sleep disturbance during recovery from critical illness after ICU discharge. The objectives were to investigate the prevalence of long-term sleep disturbance over time, summarize related factors, and identify potential measures to facilitate the prevention and treatment of long-term sleep disturbance, including that related to PICS.

## Review

Materials and methods

Protocol Registration

This scoping review was conducted following the methodology of Arksey and O'Malley [17] and the revised recommendations by Levac et al. [18]. The results were reported in accordance with the Preferred Reporting Items for Systematic Reviews and Meta-Analyses extension for Scoping Reviews (PRISMA-ScR) guidelines [19] (Appendix Table 5). This review was conducted after registering the protocol on the Open Science Framework (https://osf.io/uz3j4/).

Research Question

The review questions were as follows: (i) "What is the incidence of sleep disturbance after ICU discharge?" (ii) "What are the risk factors for sleep disturbance after ICU discharge?" (iii) "What are the PICS-related symptoms (post-traumatic stress disorder [PTSD], anxiety, chronic pain) associated with sleep disturbance after discharge in critically ill patients?" and (iv) "What interventions improve long-term sleep disturbance?"

Search Strategy

We searched the PubMed, CINAHL, and Cochrane Central Register of Controlled Trials (CENTRAL) databases from their inception until June 12, 2024. To identify sleep disturbance after ICU discharge, the following keyword combinations were used: ("Intensive care units" or "critical care" or "Critical illness" or "ICU" or "intensive care" or "critically ill" or "Acute Respiratory Failure") AND ("Sleep Wake Disorders" or "dyssomnias" or "circadian rhythm disorders" or "sleep wake disorder" or "chronobiology disorders" or "Sleep Deprivation" or "sleep disruption" or "sleep disorder" or "sleep rhythm disturbance" or "sleep disturbance" or "disturbed sleep" or "sleep disruption" or "Insomnia") AND ("after hospitalization" or "after hospitalization" or "after hospital" or "after critical illness" or "after ICU" or "post ICU" or "discharge" or "recovery" or "survivor" or "survivors" or "survivors ICU"). The complete search terms are presented in Appendix Table 6.

Eligibility Criteria

We searched for articles and employed the following inclusion criteria: (1) Population: Adult patients. (2) Concept: Incidence of sleep disturbance, their risk factors, and preventive or therapeutic interventions. (3) Context: Sleep disturbance after ICU discharge and its impact on long-term prognosis. (4) Type of Study: Articles describing long-term sleep disturbance or recovery from sleep disturbance after ICU discharge, with no restrictions on language or publication date. The following were excluded: reviews, case reports, qualitative studies, opinion pieces, letters, books, oral presentations, posters, and studies limited to abstracts.

Data Selection Process

In the first screening, the results from each database were imported into Rayyan, where the summaries (title, abstract, and full text if necessary) were reviewed, and duplicate studies were removed. Then, we divided the reviewed results among nine reviewers. Two reviewers independently assessed these summaries (title, abstract, and full text if necessary) and excluded studies that did not meet the eligibility criteria. Disagreements were resolved through discussions. In the second screening, the full texts of the selected studies were obtained and assessed to determine their eligibility. If a study was excluded, the reason for exclusion was clearly stated. Any discrepancies that arose during the literature screening process were resolved through discussions with a third reviewer.

Data Extraction and Integration

Using a customized extraction form developed by two researchers, data were collected on author names, publication year, study design, sample size, sex, sleep assessment tools, measurement time points (e.g., one month, three months, six months, 12 months, and over one year post-discharge), prevalence of sleep disturbance, risk factors for long-term sleep disturbance, and the association between long-term sleep disturbance and PICS. Prevalence cutoff points were determined as the Pittsburgh Sleep Quality Index (PSQI) score ≥5 or >5, and Insomnia Severity Index (ISI) score ≥15 or >15. Studies describing percentages using these and other assessment tools were summarized. The statistical software R (packages meta and metafor; R Foundation for Statistical Computing, Vienna, Austria) was used to calculate the prevalence of sleep disturbance and 95% confidence intervals (CIs) after ICU discharge. A random-effects meta-analysis (Der Simonian-Laird) was undertaken to compute pooled prevalence estimates and 95% CIs.

Regarding risk factors for sleep disturbance post-ICU discharge, factors that occurred before hospitalization, during ICU stay, or during the hospitalization period, and whose results were reported in a multivariable analysis, were extracted. Regarding the association between post-hospital discharge sleep disturbance and PICS, all studies in which the association was reported, regardless of the analytical method, were extracted.

In alignment with the research questions, studies on risk factors for long-term sleep disturbance, summaries of interventional studies, and studies on the association between long-term sleep disturbance and PICS were narratively integrated. The synthesis included quantitative analysis (i.e., frequency analysis) and qualitative analysis (i.e., thematic analysis) of the components of the methods to identify and display gaps in long-term sleep disturbance after ICU discharge research and conceptual definitions of gaps in long-term sleep disturbance after ICU discharge research.

Results

**Figure 1:**
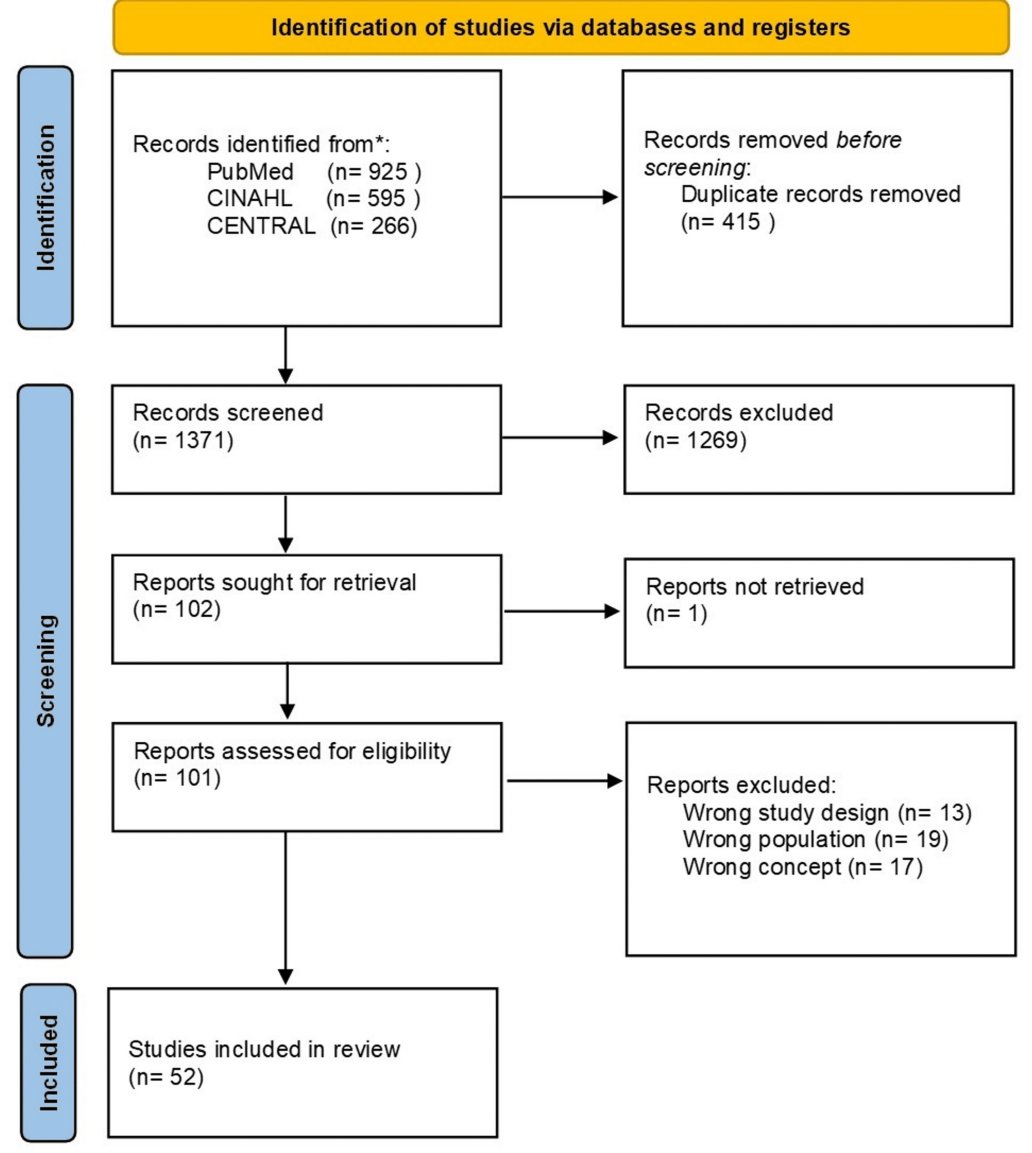
PRISMA 2020 flow diagram PRISMA, Preferred Reporting Items for Systematic Reviews and Meta-Analyses.

We initially identified 1,786 studies on sleep disturbance following ICU discharge. After excluding 415 duplicates, 1269 that were ineligible during the first screening, one that was inaccessible, and 49 that were ineligible during the second screening, a total of 52 studies were ultimately included (Figure 1).

Characteristics of Included Studies

Table 1 shows the characteristics of the included studies. The identified studies were conducted in Europe (19 studies) [20-38], North America (18 studies) [15,39-55], Asia (eight studies) [56-63], Oceania (four studies) [64-67], Latin America (two studies) [68,69], and the Middle East (one study) [70]. The study designs included 32 prospective cohort studies [20-31,33,35,36,39-43,48,51,53-56,59,64,65,68-70], two secondary analysis of prospective cohort studies [47,50], six retrospective cohort studies [38,52,57,58,60,61], four cross-sectional studies [44-46,62], one secondary analysis of cross-sectional studies [15], two randomized controlled trials (RCTs) [34,63], two secondary analyses of RCTs [32,67], two descriptive observational studies [49,66], and one quasi-experimental study [37].

**Table 1:** Characteristics of included studies Excluded from the analysis: 0; Not described or not excluded: ×; Not excluded, but the incidence of new cases is listed separately: △. ^†^Sleep disorders prior to admission. PROMIS, Patient-Reported Outcomes Measurement Information System; ISI, Insomnia Severity Index; PSQI, Pittsburgh Sleep Quality Index; GSDS, General Sleep Disturbance Scale; SCL, Symptom Checklist; RCSQ, Richards-Campbell Sleep Questionnaire; ESS, Epworth Sleepiness Scale; SICQ, Sleep in the Intensive Care Unit Questionnaire; EORTC, European Organisation for Research and Treatment of Cancer; PCL, Post-Traumatic Stress Disorder; 15D, 15-dimensional, standardized, self-administered measure of health-related quality of life; BNSQ, Basic Nordic Sleep Questionnaire; RGSQ, Reduced Global Sleep Quality Score; N/A, not applicable.

Authors, year	Country	Setting	Study design	Sleep disorders^†^	Measurement instruments	
					Objective	Subjective
Khan et al., 2024 [63]	India	Acute respiratory failure	RCT	×	N/A	PROMIS Short Form
Berger et al., 2023 [29]	France	COVID-19	Prospective cohort	×	N/A	ISI
Rousseau et al., 2023 [31]	France	COVID-19	Prospective cohort	△	N/A	PSQI
Jain et al., 2023 [62］	India	COVID-19	Cross-sectional	〇	N/A	PSQI
Georgopoulos et al., 2023 [38］	Greece	Medical-surgical ICU	Prospective cohort	〇	PSG	N/A
Galeoto et al., 2023 [35］	Italy	Hip and knee replacement	Prospective cohort	△	N/A	GSDS
Guillen-Burgos et al., 2023 [69］	Colombia	COVID-19	Prospective cohort	×	N/A	ISI
Neville et al., 2022 [42］	USA	COVID-19	Prospective cohort	×	N/A	PROMIS
Benítez et al., 2022 [22］	Spain	COVID-19	Prospective cohort	×	Actigraphy	PSQI
Claus et al., 2022 [23］	Belgium	COVID-19	Prospective cohort	△	N/A	ISI
Oh et al., 2022 [57」	Korea	Acute respiratory distress syndrome	Retrospective cohort	△	N/A	ICD-10 codes
Ko et al., 2022 [61］	Korea	Medical-surgical ICU	Retrospective cohort	×	N/A	ICD-10 codes
Clemente et al., 2022 [36］	Italy	COVID-19	Prospective cohort	×	N/A	SCL
Park et al., 2022 [58］	South Korea	ECMO	Retrospective cohort	△	N/A	ICD-10 codes
Wilcox et al., 2021 [39］	Canada	Medical-surgical ICU	Prospective cohort	〇	Actigraphy	N/A
Rousseau et al., 2021 [20］	Belgium	COVID-19	Prospective cohort	×	N/A	PSQI
Srikanth et al., 2021 [56]	India	Acute respiratory distress syndrome	Prospective cohort	〇	PSG	PSQI, ISI, RCSQ, ESS
Elías et al., 2021 [45]	USA	Level 1 trauma	Cross-sectional	×	Actigraphy	N/A
Martillo et al., 2021 [49]	USA	COVID-19	Descriptive cohort study	×	N/A	ISI
Shang et al., 2021 [59]	China	COVID-19	Prospective cohort	×	N/A	Interviews
Yang et al., 2020 [43]	USA	Acute respiratory failure	Prospective cohort	×	Actigraphy	ISI
Wilcox et al., 2019 [40]	Canada	ICU	Prospective cohort	△	Actigraphy	PSQI, RCSQ
Wang et al., 2019 [44]	USA	Critical care recovery clinic	Cross-sectional	△	N/A	Original questionnaire
Alexopoulou et al., 2019 [21]	Greece	Medical-surgical ICU	Prospective cohort	〇	PSG	N/A
Caruana et al., 2018 [65]	Australia	Open-heart surgery	Prospective cohort	×	N/A	PSQI, ISI, RCSQ, SICQ
Langerud et al., 2018 [24]	Norway	Medical-surgical ICU	Prospective cohort	×	N/A	GSDS
Parsons et al., 2018 [47]	USA	Medical-surgical ICU	Secondary analysis of prospective cohort	×	N/A	ISI
Zhang et al., 2018 [60]	China	ICU	Retrospective cohort	〇	PSG	PSQI
Altman et al., 2018 [51]	USA	Medical ICU	Prospective cohort	×	N/A	PSQI
Elías, 2018 [54]	USA	Medical-surgical ICU	Prospective cohort	×	Actigraphy	N/A
Rodríguez-Villar et al., 2017 [30]	Spain	Multiorgan failure	Prospective cohort	×	N/A	N/A
Schädler et al., 2017 [32]	Germany	Ventilation	Secondary analysis of RCT	×	N/A	EORTC C30
Scarpa et al., 2017 [34]	Italy	Undergoing esophagectomy	RCT	×	N/A	PSQI, EORTC C30
Solverson et al., 2016 [41]	Canada	Medical-surgical ICU	Prospective cohort	×	Actigraphy	PSQI, ESS
Parsons et al., 2015 [15]	USA	Medical-surgical ICU	Secondary analysis of cross-sectional study	×	N/A	ISI
Fischer et al., 2015 [52]	USA	Surgical ICU	Retrospective cohort	×	N/A	EORTC C30
McKinley et al., 2013 [64]	Australia	General ICU	Prospective cohort	〇	N/A	PSQI, ISI, RCSQ
Choi et al., 2014 [48]	USA	Medical ICU	Prospective cohort	×	N/A	PSQI
Vesz et al., 2013 [68]	Brazil	Medical-surgical ICU	Prospective cohort	×	N/A	ESS
Parsons et al., 2012 [50]	USA	Acute lung injury	Secondary analysis of prospective cohort	×	N/A	PCL
McKinley et al., 2012 [67]	Australia	Medical-surgical ICU	Secondary analysis of RCT	×	N/A	15D
Van Gulik et al., 2011 [26]	Netherland	Cardiac surgery	Prospective cohort	×	N/A	Original questionnaire
Lee et al., 2009 [46]	USA	Acute respiratory distress syndrome	Cross-sectional	〇	PSG	ISI, ESS
Kelly and McKinley 2010 [66]	Australia	Medical-surgical ICU	Descriptive study	×	N/A	Original questionnaire
Cronberg et al., 2009 [33]	Sweden	Cardiac arrest and therapeutic hypothermia	Prospective cohort	×	N/A	Skane Sleep Index
Orwelius et al., 2008 [27]	Sweden	Medical-surgical ICU	Prospective cohort	△	N/A	BNSQ
Diby et al., 2008 [37]	Swiss	Elective cardiac surgery	Quasi-experimental	×	N/A	PSQI, RGSQ
BaHammam, 2006 [70]	Saudi Arabia	Acute myocardial infarction	Prospective cohort	〇	PSG	N/A
Granja et al., 2005 [28]	Portugal	Medical-surgical ICU	Prospective cohort	×	N/A	Original questionnaire
Dimopoulou et al., 2001 [53]	USA	Cardiac surgery	Prospective cohort	×	N/A	Original questionnaire
Eddleston et al., 2000 [25]	UK	Acute general surgery	Prospective cohort	×	N/A	Original questionnaire
Simpson et al., 1996 [55]	USA	Emergency cardiac surgery	Prospective cohort	〇	N/A	N/A

The follow-up periods for long-term sleep disturbance were less than one month to three months for 16 studies [20,22,31,34-36,40,41,43-45,49,54,56,60,68], less than four months to six months for 16 studies [21,28,33,37,38,42,46,48,50,51,59,64-67,70], over seven months to 12 months for 14 studies [15,23-27,29,30,32,39,47,57,58,63], between one and two years for one study [62], two years for two studies [61,69], four years for one study [53], and six years for one study [52]. Regarding exclusion criteria, 10 studies [21,38,39,46,55,56,60,62,64,70] excluded participants with sleep disturbance before hospitalization, eight studies [23,27,31,35,40,44,57,58] excluded participants during analysis, and 34 studies either had no exclusion criteria or included all participants in the analysis.

Prevalence of Sleep Disturbance After Hospitalization

**Figure 2:**
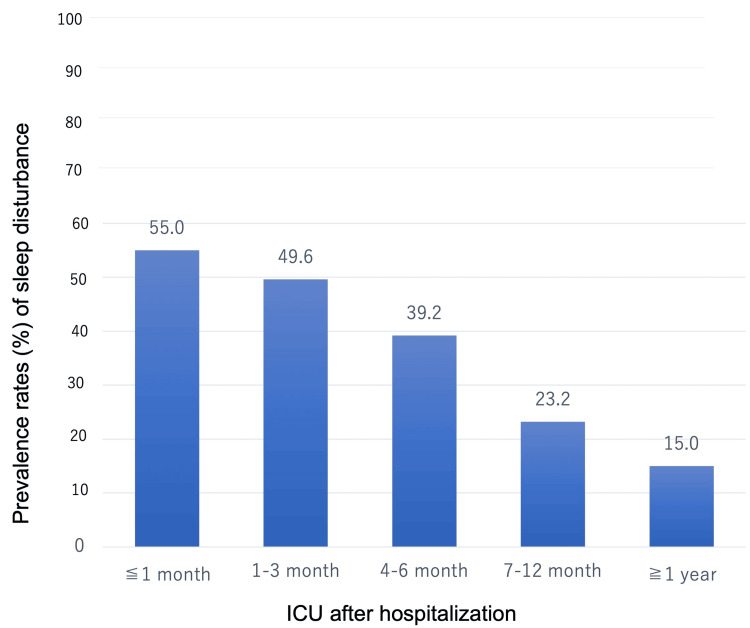
Prevalence of sleep disturbance after hospitalization ICU, intensive care unit.

Prevalence of sleep disturbance was calculated from 42 studies [15,20,22-31,33-38,40,41,43,44,46-54,56-59,61,64-69] that showed sleep disturbance rates (Figure 2). Prevalence was measured at five time points: less than one month after discharge in 12 studies [34,35,37,38,40,48,49,54,56,65,67,68], 1-3 months in 13 studies [20,22,24,25,31,36,41,43,44,48,56,64,65], 4-6 months in 13 studies [23,27,33,37,46,48,50,51,59,66,67], 7-12 months in 10 studies [15,24-26,28-30,47,57,58], and over one year in four studies [52,53,61,69]. The sleep disturbance measurement tools used after discharge included the PSQI (n = 14) [20,22,31,34,37,40,41,48,51,56,60,62,64,65], ISI (n = 11) [15,23,29,43,46,47,49,56,64,65,69], the Richards-Campbell Sleep Questionnaire (RCSQ) (n = 4) [40,56,64,65], Epworth Sleepiness Scale (ESS) (n = 4) [41,46,56,68], International Classification of Diseases-10th Revision (ICD-10) codes (n = 3) [57,58,61], European Organisation for Research and Treatment of Cancer Questionnaire (EORTC-C30) (n = 3) [32,52], and other evaluation tools (n = 20) [24-28,33-37,42,44,50,52,53,59,63,65-67]. The prevalence of long-term sleep disturbance at each time point was as follows: less than one month after ICU discharge, 55.0% (95% CI: 42.8-66.6); 1-3 months, 49.6% (95% CI: 42.3-57.0); 4-6 months, 39.2% (95% CI: 27.5-52.4); 7-12 months, 23.2% (95% CI: 14.7-34.8); and over one year, 15.0% (95% CI: 7.5-27.5).

Risk Factors for Sleep Disturbance After Hospitalization

Risk factors for sleep disturbance were extracted based on those found to be significant in multivariate analysis (Table 2). The main pre-hospital risk factors were older age [51], female sex [27,59], prehospital sleep disturbance [64,65], and presence of concurrent diseases, such as diabetes mellitus (DM) and cancer [27]. ICU-related factors included 22 duration of mechanical ventilation [30,56], and duration of sedation or analgesia [56]. Hospital-wide factors, including both ICU and general wards, included high illness severity [21], pain [24], delirium [57], increased duration of delirium [51], poor grip strength and dexterity [45,54], ICU and hospital lengths of stay [30,56], and poor sleep quality in the hospital ward [64]. Intervention focuses included shortening the duration of mechanical ventilation, shortening sedation or analgesia duration, pain relief, delirium prevention, and ensuring good quality sleep in the hospital ward.

**Table 2:** Risk factors for sleep disturbance in long-term ICU survivors DM, diabetes mellitus; ICU, intensive care unit; V-A ECMO, veno-arterial extracorporeal membrane oxygenation.

	Risk factors
Pre-hospital	Older age [51], female sex [27,59], prehospital sleep disturbances [64,65], preexisting psychiatric illness: anxiety [57], depression [51,57], substance abuse [57], concurrent diseases (for example, DM and cancer) [27], underlying disability (mild to moderate-severe) [58]
Treatments in ICU	Longer mechanical ventilation days [30,56], duration of sedation or analgesia [56], V-A ECMO [58]
In hospital	Pain [24], delirium [57], increasing delirium days [51], sepsis [40], renal failure [30], higher severity of illness [21], worse grip strength and dexterity [45,54], ICU and hospital length of stay [30,56], poor sleep quality in the hospital ward [64]

Association Between PICS and Sleep Disturbance After Hospitalization

As shown in Table 3, the association between long-term sleep disturbance and PICS that was significant in univariate analysis was reported in 19 articles [15,21-23,27,28,30,39,41,44,47,50,51,58,61,62,64,65,67]. Sleep disturbance was associated with all aspects of PICS, including physical disability assessed by Modified Rankin Scale [30], cognitive impairment [39,61], mental health [15,22,23,41,44,47,51,58,62,64,65,67], and quality of life (QOL) [15,21,27,28,41,47,50,64,65,67]. The most common association was with depression in 10 studies [15,22,44,47,51,58,62,64,65,67], followed by that with PTSD in seven studies [15,23,44,47,58,64,65], and anxiety in six studies [22,41,62,64,65,67]. Although most studies of mental dysfunction were conducted within one year, depression was associated with long-term effects in a study lasting longer than a year [62]. For QOL, sleep disturbance was associated with physical function [15,47,50], role-physical [27,47], bodily pain [15,27,47], general health [27,47,50], vitality [15,27,47,50], social functioning [47], role-emotional [15], and mental health [27,47,50]. The most common evaluation periods ranged from four to six months. Cognitive impairment was also associated with long-term effects in a study that lasted over a year [61].

**Table 3:** Results of the association between PICS and sleep disturbance For health-related QOL, sleep disturbances were associated with physical functioning, role physical, bodily pain, general health, vitality, social functioning, role-emotional, and mental health. PICS, post-intensive care syndrome; PTSD, post-traumatic stress disorder; QOL, quality of life; HRQOL, health-related quality of life.

PICS		Timing of survey
Physical	Physical disability assessed by Modified Rankin Scale [30]	6 months after discharge [30]
Cognitive	Cognitive impairment [39,61]	≦1 month after discharge [39], ≧12 months after discharge [61]
Mental health	Psychological distress [15,22,47,51,58,62,64,65,67], anxiety [22,41,62,64,65,67], depression [15,22,44,47,51,58,62,64,65,67], PTSD [15,23,44,47,58,64,65], stress [64,65,67]	≦1 month after discharge [67], 1-3 months after discharge [22,41,44,67], 4-6 months after discharge [23,64,65,67], 7-12 months after discharge [15,47,51,58], ≧12 months after discharge [62]
Quality of life	Lower physical and mental HRQOL [15,27,28,41,47,50,64,67], physical function [15,47,50], role physical [27,47], bodily pain [15,27,47], general health [27,47,50], vitality [15,27,47,50], social functioning [47], role-emotional [15,47], mental health [27,47,50], PCS (physical component summary) [21,41,50,65], MCS (mental component summary) [15,41,50,65,67]	≦1 month after discharge [21], 1-3 months after discharge [41], 4-6 months after discharge [27,28,50,64,65,67], 7-12 months after discharge [15,47]

Intervention for Sleep Disturbance After Hospitalization

Only two RCTs [34,63] focused on interventions for prevention and treatment. The results are shown in Table 4. One study [34] was a prevention intervention targeting after-surgery patients. The preventive intervention conducted in the ICU combined psychological counseling with sleep aids such as earplugs and eye masks. In another study [63], therapeutic interventions were conducted after hospital discharge. Sleep disturbances were assessed at various time points. Results of the intervention studies showed that psychological counseling [34] and a 12-month nurse-led collaborative care intervention [63] were effective in improving long-term sleep disturbances. However, two studies required intervention by psychologists and other professionals. The use of sleep aids such as earplugs and eye masks had no effect on sleep quality or duration of sleep medication use at discharge [34].

**Table 4:** Intervention for long-term sleep disturbance after ICU discharge: RCTs ICU, intensive care unit; RCT, randomized controlled trial; CBT-I, cognitive behavioral therapy for insomnia; PSQI, Pittsburgh Sleep Quality; TST, total sleep time; SE, sleep efficacy; PSAM, psychological counselling + sleep adjuvant measures; PC, psychological counselling; SAM, sleep adjuvant measures; m-CCRP, Mobile Critical Care Recovery Program; ARF, acute respiratory failure.

Authors, year	Objective	Methods of intervention	Timing of intervention/Patient	Number of interventions	Control	Sleep disturbance		Results
						Intervention group	Control	
Scarpa et al., 2017 [34]	To evaluate the effect of psychological counselling and sleep adjuvant measures on postoperative quality of sleep and quality of life	Three-arm intervention: PSAM: PC + SAM; PC by psychologist; SAM: Earplugs and eye masks	ICU and surgical ward/oesophagectomy	① 30 min on admission; ② 15 min on postoperative day 1; ③ 30 min in the surgical ward; ④ 30 min in the surgical ward before discharge	Standard care (not earplugs and eye masks)	PSQI, C30-SL; PSAM: 4/16; PC: 7/19; SAM: 10/19	14/20	PC reduced the impairment of quality of sleep
Khan et al., 2024 [63]	To evaluate the efficacy of a post-ICU program in improving the QOL of ARF survivors	m-CCRP: The care coordinator conducted patient visits at both home and healthcare facilities, collaborated with the m-CCRP interdisciplinary team, maintained communication with the patient’s primary care provider and specialists, and implemented the individualized care plans	After hospital discharge/ARF	Every 2 weeks for the first 6 months and once per month for the last 6 months	Telephone-based control	PROMIS Sleep Disturbance Short Form 4a 26/233	21/233	M-CCRP improved sleep scores

Discussion

In this scoping review, 52 studies on long-term sleep disturbance in recovery from critical illness were selected, and approximately half of the patients experienced sleep disturbance post-ICU discharge, decreasing to 15% after one year or more. The risk factors for sleep disturbance were long duration of mechanical ventilation [30,56], long duration of sedation or analgesia [56], high severity of illness [21], pain [24], delirium [51,57], and poor grip strength and dexterity [45,54]. Sleep disturbance was associated with PICS, including physical disability [30], cognitive impairment [39,61], mental health [15,22,23,41,44,47,51,58,62,64,65,67], and QOL [15,21,27,28,41,47,50,64,65,67]. Only two intervention studies [34,63] were focused on the prevention and treatment of long-term sleep disturbance.

The prevalence of sleep disturbance after ICU discharge decreased over time. A prior meta-analysis on the dynamic prevalence of sleep disturbance in critically ill patients [9] revealed rates of 64% at two months, 49% at three months, 40% at six months, and 28% at 12 months following ICU discharge. A systematic review [16] revealed rates of 50-66.7% at one month after ICU discharge, 34-64.3% at 1-3 months, 22-57% at 3-6 months, and 10-61% beyond six months. Similar to the findings of the present study, these results showed that the highest prevalence was immediately after discharge from the ICU, and that sleep disturbance decreased over time. Additionally, 90% of data on long-term sleep disturbance were obtained from follow-ups of one year or less, and there was a lack of longer-term data, suggesting the need for further long-term follow-up studies.

Risk factors for long-term sleep disturbance included pre-hospital factors such as older age [51], female sex [27,59], prehospital sleep disturbance [64,65], and presence of concurrent diseases, such as DM and cancer [27], as well as ICU treatment factors such as long duration of mechanical ventilation [30,56], long duration of sedation or analgesia [56], high severity of illness [21], long durations of ICU and hospital stay [56], and poor sleep quality in the hospital ward [64]. These factors were consistent with findings from a previous systematic review of sleep disturbance after hospital discharge in critically ill patients [16]. However, this previous review excluded postoperative patients who underwent major surgery. Therefore, the current review, which included articles on surgical patients, identified new risk factors such as pain [24], delirium [57], increased delirium duration [51], sepsis [40], renal failure [30], high illness severity [21], and poor grip strength and dexterity [45,54]. Risk factors that can be modified from length of ICU stay are prolonged mechanical ventilation, prolonged sedation or analgesic dosage, pain, delirium, poor grip strength and dexterity, and poor sleep quality on the ward. Identification of these risk factors and implementation of preventive interventions during the early stages of ICU stay may be a focus for preventing long-term sleep disturbance.

Sleep disturbance in ICU survivors has been reported as a new condition related to PICS [11], and the results of the present study support this finding. Although the prevalence of sleep disturbance decreases over time post-discharge, it remains associated with PICS symptoms and low QOL, leading to long-term negative impacts. Therefore, the results of this study suggest the potential benefit of sleep disorder interventions as part of a long-term recovery program for patients six months to ≥1 year after ICU discharge, along with interventions for physical, cognitive, and mental health dysfunctions as part of PICS. In addition, some studies involved follow-up periods of 1.5 years [62] and five years [12] after intensive care, to assess HRQOL. However, no follow-up studies have been conducted on the association between sleep disturbance and PICS after ICU discharge for longer than one year, suggesting the need for longer-term follow-ups.

Only two intervention studies [34,63] on long-term sleep disturbance were found, all of which involved programs conducted by specialists such as clinical psychologists. These interventions are not feasible in facilities without such professionals. In addition, the efficacy of brief interventions, including earplugs, eye masks, and adjustments to the sleep environment in ICUs and hospital wards, has been insufficiently studied, and their impact on long-term sleep disturbance remains unclear. Furthermore, no intervention studies have been conducted on the long-term effects of good symptom management (such as light sedation, pain control, and delirium prevention) in ICU settings on long-term sleep disturbance. Therefore, further research is required on these comprehensive interventions for sleep disturbance.

Strengths and Limitations of This Review

Our scoping review included postoperative patients, unlike previous reviews [16], and integrated results of studies on various ICU patients. Furthermore, preventive and therapeutic interventions for long-term sleep disturbance were analyzed, addressing specific intervention methods for these conditions. The prevalence of long-term sleep disturbance and its association with PICS was also clarified, highlighting the necessity for long-term medical interventions. This research could potentially enhance understanding of sleep disturbance after ICU discharge and emphasize the need for medical interventions.

However, this study has some limitations. The prevalence of sleep disturbance was not validated against objective sleep parameters, such as polysomnography or actigraphy. Instead, it was evaluated using the PSQI and ISI, which are subjective tools and may be influenced by individual perceptions. In addition, no formal bias assessment was conducted; therefore, the reported prevalence and identified risk factors should be interpreted cautiously. Moreover, only risk factors adjusted for confounding variables through multivariate analysis in each study were extracted, which may not account for all potential biases. Sleep disturbance after ICU discharge is often overlooked due to a complex interplay of factors. Therefore, there is an urgent need to identify high-risk patients.

## Conclusions

The prevalence of sleep disturbance after ICU discharge in critically ill patients is very high, with over half suffering from sleep disturbance within one month of discharge, although recovery may occur, with around 15% improving within the following year. There is an indication of a link between PICS and post-discharge sleep disturbance; however, preventive intervention studies on long-term sleep disorder risk factors are lacking. Further research on comprehensive interventions for sleep disturbance, addressing physical function and mental health, is required.

## Appendices

**Table 5:** The Preferred Reporting Items for Systematic Reviews and Meta-Analysis 2020 Checklist

Section and Topic	Item #	Checklist item	Location where item is reported
TITLE			
Title	1	Identify the report as a systematic review.	P. 1
ABSTRACT			
Abstract	2	See the PRISMA 2020 for Abstracts checklist.	P. 2
INTRODUCTION			
Rationale	3	Describe the rationale for the review in the context of existing knowledge.	P. 2
Objectives	4	Provide an explicit statement of the objective(s) or question(s) the review addresses.	P. 2
METHODS			
Eligibility criteria	5	Specify the inclusion and exclusion criteria for the review and how studies were grouped for the syntheses.	P. 3
Information sources	6	Specify all databases, registers, websites, organizations, reference lists, and other sources searched or consulted to identify studies. Specify the date when each source was last searched or consulted.	P. 3
Search strategy	7	Present the full search strategies for all databases, registers, and websites, including any filters and limits used.	P. 3, Table 1
Selection process	8	Specify the methods used to decide whether a study met the inclusion criteria of the review, including how many reviewers screened each record and each report retrieved, whether they worked independently, and if applicable, details of automation tools used in the process.	P. 3
Data collection process	9	Specify the methods used to collect data from reports, including how many reviewers collected data from each report, whether they worked independently, any processes for obtaining or confirming data from study investigators, and if applicable, details of automation tools used in the process.	P. 3
Data items	10a	List and define all outcomes for which data were sought. Specify whether all results that were compatible with each outcome domain in each study were sought (e.g. for all measures, time points, analyses), and if not, the methods used to decide which results to collect.	P. 3–４
	10b	List and define all other variables for which data were sought (e.g. participant and intervention characteristics, funding sources). Describe any assumptions made about any missing or unclear information.	P. 3–４
Study risk of bias assessment	11	Specify the methods used to assess risk of bias in the included studies, including details of the tool(s) used, how many reviewers assessed each study and whether they worked independently, and if applicable, details of automation tools used in the process.	P. 3–４
Effect measures	12	Specify for each outcome the effect measure(s) (e.g. risk ratio, mean difference) used in the synthesis or presentation of results.	P. 3–４
Synthesis methods	13a	Describe the processes used to decide which studies were eligible for each synthesis (e.g. tabulating the study intervention characteristics and comparing against the planned groups for each synthesis (item #5)).	P. 3
	13b	Describe any methods required to prepare the data for presentation or synthesis, such as handling of missing summary statistics, or data conversions.	N/A
	13c	Describe any methods used to tabulate or visually display results of individual studies and syntheses.	P.４
	13d	Describe any methods used to synthesize results and provide a rationale for the choice(s). If meta-analysis was performed, describe the model(s), method(s) to identify the presence and extent of statistical heterogeneity, and software package(s) used.	P. 3–４
	13e	Describe any methods used to explore possible causes of heterogeneity among study results (e.g. subgroup analysis, meta-regression).	N/A
	13f	Describe any sensitivity analyses conducted to assess robustness of the synthesized results.	N/A
Reporting bias assessment	14	Describe any methods used to assess risk of bias due to missing results in a synthesis (arising from reporting biases).	N/A
Certainty assessment	15	Describe any methods used to assess certainty (or confidence) in the body of evidence for an outcome.	N/A
RESULTS			
Study selection	16a	Describe the results of the search and selection process, from the number of records identified in the search to the number of studies included in the review, ideally using a flow diagram.	P.4 Figure 1
	16b	Cite studies that might appear to meet the inclusion criteria, but which were excluded, and explain why they were excluded.	N/A
Study characteristics	17	Cite each included study and present its characteristics.	P.4 Table 2
Risk of bias in studies	18	Present assessments of risk of bias for each included study.	N/A
Results of individual studies	19	For all outcomes, present, for each study: (a) summary statistics for each group (where appropriate) and (b) an effect estimate and its precision (e.g. confidence/credible interval), ideally using structured tables or plots.	P. 4–6
Results of syntheses	20a	For each synthesis, briefly summarize the characteristics and risk of bias among contributing studies.	N/A
	20b	Present results of all statistical syntheses conducted. If meta-analysis was done, present for each the summary estimate and its precision (e.g. confidence/credible interval) and measures of statistical heterogeneity. If comparing groups, describe the direction of the effect.	P. 4–6
	20c	Present results of all investigations of possible causes of heterogeneity among study results.	N/A
	20d	Present results of all sensitivity analyses conducted to assess the robustness of the synthesized results.	N/A
Reporting biases	21	Present assessments of risk of bias due to missing results (arising from reporting biases) for each synthesis assessed.	N/A
Certainty of evidence	22	Present assessments of certainty (or confidence) in the body of evidence for each outcome assessed.	N/A
DISCUSSION			
Discussion	23a	Provide a general interpretation of the results in the context of other evidence.	P. 6
	23b	Discuss any limitations of the evidence included in the review.	P. 7
	23c	Discuss any limitations of the review processes used.	P. 7
	23d	Discuss implications of the results for practice, policy, and future research.	P. 6–7
OTHER INFORMATION			
Registration and protocol	24a	Provide registration information for the review, including register name and registration number, or state that the review was not registered.	N/A
	24b	Indicate where the review protocol can be accessed, or state that a protocol was not prepared.	N/A
	24c	Describe and explain any amendments to information provided at registration or in the protocol.	N/A
Support	25	Describe sources of financial or non-financial support for the review, and the role of the funders or sponsors in the review.	P. 12-13
Competing interests	26	Declare any competing interests of review authors.	P. 13
Availability of data, code, and other materials	27	Report which of the following are publicly available and where they can be found: template data collection forms; data extracted from included studies; data used for all analyses; analytic code; any other materials used in the review.	N/A

**Table 6:** Search terms

Database	Search terms
PubMed	#1 "Intensive care units"[mh] OR "critical care"[mh] OR "Critical illness"[mh] OR ICU[tiab] OR "intensive care unit*"[tiab] OR "intensive care"[tiab] OR "critical care"[tiab] OR "critical illness"[tiab] OR "critically ill"[tiab] OR "Acute Respiratory Failure"[tiab] #2 "Sleep Wake Disorders*"[mh] OR dyssomnias[mh] OR "circadian rhythm disorders"[tiab] OR "sleep wake disorder*"[tiab] OR "Dyssomnias"[tiab] OR "Chronobiology Disorders"[tiab] OR "Sleep Deprivation"[tiab] OR "sleep disruption"[tiab] OR "sleep disorder*"[tiab] OR "sleep rhythm disturbance*"[tiab] OR "sleep disturbance*"[tiab] OR "disturbed sleep"[tiab] OR "sleep disruption" [tiab] Insomnia[tiab] #3 "after hospitalization"[tiab] OR "after hospitalisation"[tiab] OR "after hospital"[tiab] OR "after critical illness "[tiab] OR "after ICU"[tiab] OR "post ICU"[tiab] OR discharge[tiab] OR recovery[tiab] OR survivor*[tiab] OR survivors [tiab] OR survivors [mh] #1 AND #2 AND #3
Cochrane CENTRAL	#1 MeSH descriptor:[Intensive Care Units]explode all trees OR MeSH descriptor:[Critical Care]explode all trees OR MeSH descriptor:[Critical illness]explode all trees OR ICU, "intensive care unit" OR "critical illness" OR "critically ill" OR "Acute Respiratory Failure" #2 MeSH descriptor:[Sleep Wake Disorders]explode all trees OR MeSH descriptor:[dyssomnias]explode all trees OR "circadian rhythm disorders" OR "sleep wake disorder" OR "Chronobiology Disorders" OR "Sleep Deprivation" OR "sleep disruption" OR "sleep disorder" OR "sleep rhythm disturbance" OR "sleep disturbance" OR "disturbed sleep" OR "sleep disruption" OR Insomnia #3 "after hospitalization" OR "after hospitalisation" OR "after hospital" OR "after critical illness " OR "after ICU" OR "post ICU" OR discharge OR recovery OR survivor* OR MeSH descriptor:[survivors ]explode all trees #1 AND #2 AND #3
CINAHL	#1 MH Intensive care units OR MH critical care OR MH Critical illness OR TI(ICU) OR AB (ICU) OR TI("intensive care unit*") OR AB ("intensive care unit*") OR TI("intensive care") OR AB ("intensive care") OR TI("critical care") OR AB ("critical care") OR TI("critical illness") OR AB ("critical illness") OR TI("critically ill") OR AB ("critically ill") OR TI("Acute Respiratory Failure" ) OR AB ("Acute Respiratory Failure" ) #2 MH dyssomnias OR TI ("circadian rhythm disorders" )OR AB ("circadian rhythm disorders") OR TI( "sleep wake disorder*" )OR AB( "sleep wake disorder*") OR TI(dyssomnias)OR AB(dyssomnias) OR TI("Sleep Deprivation") OR AB("Sleep Deprivation") OR TI("sleep disruption") OR AB("sleep disruption") OR TI("sleep disorder*") OR AB("sleep disorder*") OR TI("sleep disturbance*") OR AB("sleep disturbance*") OR TI("disturbed sleep") OR AB("disturbed sleep") OR TI("sleep disruption" ) OR AB("sleep disruption" ) OR TI(Insomnia) OR AB(Insomnia) #3 TI("after hospitalization") OR AB("after hospitalization") OR TI("after hospitalisation") OR AB("after hospitalisation") OR TI("after hospital" ) OR AB("after hospital") OR TI("after critical illness ") OR AB("after critical illness " ) OR TI("after ICU" ) OR AB("after ICU") OR TI("post ICU") OR AB("post ICU" ) OR TI(discharge) OR AB(discharge) OR TI(recovery ) OR AB(recovery) OR TI(survivor*) OR AB(survivor* ) OR TI(survivors ) OR AB(survivors ) OR MH survivors #1 AND #2 AND #3
